# Identification of a naturally-occurring canine model for early detection and intervention research in high grade urothelial carcinoma

**DOI:** 10.3389/fonc.2022.1011969

**Published:** 2022-11-11

**Authors:** Deepika Dhawan, José A. Ramos-Vara, Sagar M. Utturkar, Audrey Ruple, Sarah A. Tersey, Jennifer B. Nelson, Bruce R. Cooper, Hock Gan Heng, Elaine A. Ostrander, Heidi G. Parker, Noah M. Hahn, Larry G. Adams, Christopher M. Fulkerson, Michael O. Childress, Patty L. Bonney, Christine Royce, Lindsey M. Fourez, Alexander W. Enstrom, Lisbeth A. Ambrosius, Deborah W. Knapp

**Affiliations:** ^1^ Department of Veterinary Clinical Sciences, College of Veterinary Medicine, Purdue University, West Lafayette, IN, United States; ^2^ Department of Comparative Pathobiology, College of Veterinary Medicine, Purdue University, West Lafayette, IN, United States; ^3^ Purdue University Center for Cancer Research, West Lafayette, IN, United States; ^4^ Department of Public Health, College of Health and Human Sciences, Purdue University, West Lafayette, IN, United States; ^5^ Department of Medicine, Section of Endocrinology, Metabolism, and Diabetes, University of Chicago, Chicago, IL, United States; ^6^ Bindley Bioscience Center, Purdue University, West Lafayette, IN, United States; ^7^ National Human Genome Research Institute, National Institutes of Health, Bethesda, MD, United States; ^8^ Department of Oncology and Urology, Johns Hopkins University School of Medicine, and Sidney Kimmel Comprehensive Cancer Center, Baltimore, MD, United States

**Keywords:** animal models, bladder cancer, cancer prevention, cancer screening, dog, early intervention, transitional cell carcinoma, urothelial carcinoma

## Abstract

**Background:**

Early detection and intervention research is expected to improve the outcomes for patients with high grade muscle invasive urothelial carcinoma (InvUC). With limited patients in suitable high-risk study cohorts, relevant animal model research is critical. Experimental animal models often fail to adequately represent human cancer. The purpose of this study was to determine the suitability of dogs with high breed-associated risk for naturally-occurring InvUC to serve as relevant models for early detection and intervention research. The feasibility of screening and early intervention, and similarities and differences between canine and human tumors, and early and later canine tumors were determined.

**Methods:**

STs (n=120) ≥ 6 years old with no outward evidence of urinary disease were screened at 6-month intervals for 3 years with physical exam, ultrasonography, and urinalysis with sediment exam. Cystoscopic biopsy was performed in dogs with positive screening tests. The pathological, clinical, and molecular characteristics of the “early” cancer detected by screening were determined. Transcriptomic signatures were compared between the early tumors and published findings in human InvUC, and to more advanced “later” canine tumors from STs who had the typical presentation of hematuria and urinary dysfunction. An early intervention trial of an oral cyclooxygenase inhibitor, deracoxib, was conducted in dogs with cancer detected through screening.

**Results:**

Biopsy-confirmed bladder cancer was detected in 32 (27%) of 120 STs including InvUC (n=29, three starting as dysplasia), grade 1 noninvasive cancer (n=2), and carcinoma *in situ* (n=1). Transcriptomic signatures including druggable targets such as EGFR and the PI3K-AKT-mTOR pathway, were very similar between canine and human InvUC, especially within luminal and basal molecular subtypes. Marked transcriptomic differences were noted between early and later canine tumors, particularly within luminal subtype tumors. The deracoxib remission rate (42% CR+PR) compared very favorably to that with single-agent cyclooxygenase inhibitors in more advanced canine InvUC (17-25%), supporting the value of early intervention.

**Conclusions:**

The study defined a novel naturally-occurring animal model to complement experimental models for early detection and intervention research in InvUC. Research incorporating the canine model is expected to lead to improved outcomes for humans, as well as pet dogs, facing bladder cancer.

## 1 Introduction

The need for better cancer therapies is unquestioned, yet nine of ten new cancer drugs fail during phase I, II, and III human clinical trials (1–3). An important reason for this dismal success rate is that experimental animal models used in drug development research often fail to adequately represent human cancer (4–7). This is especially the case when considering the complexities of human cancer such as spontaneous nature, cancer heterogeneity, multifaceted tumor microenvironment, drug resistance molecular pathways, aggressive and metastatic cancer behavior, variable immunologic responses, and intricate tumor-host interactions (4–7). While experimental cancer models used in carefully controlled studies will continue to be essential to assess the toxicology, mechanisms, and initial antitumor activity of cancer drugs, there is a crucial need for additional complementary models that more closely represent human cancer. Pet dogs with specific forms of naturally-occurring cancer are rapidly emerging as complementary animal models for cancer treatment research as canine cancer more closely mimics human cancer in pathological, cellular, and molecular features; cancer heterogeneity; aggressive metastatic behavior; host immunocompetence; and treatment response (8–24). Dogs are also large enough for procedures such as those involving surgery, endoscopy, various imaging modalities, and the evaluation of medical devices. With the estimated four million pet dogs that newly develop cancer each year in the US, there are ample numbers of dogs to participate in clinical studies (8). Clinical studies involving pet dogs are generally well accepted as each dog is expected to benefit, and new knowledge is gained to ultimately improve the outlook for humans, as well as pet dogs, facing cancer (8).

In addition to the need for animal models of locally-advanced and metastatic cancer, there is growing recognition for the need for relevant animal models for early cancer detection and early intervention research, i.e. models to test therapies in the earliest detectable stages of cancer. Early stage cancer is expected to respond much more favorably to therapy than advanced cancer, and early intervention represents an important area for advancement in cancer therapy (25–27). Compared to later stage cancer, early-stage disease is associated with less genetic diversity, fewer active drug resistance pathways, more competent host antitumor immune responses, and adequate tumor blood supply to facilitate drug delivery (25–27). In addition, patients with a lower cancer burden are expected to be healthier and better able to handle various forms of cancer therapy. Early intervention opens the door for the use of drugs that could have an important role in early cancer, even if those drugs are much less effective against advanced cancer. Research to advance early intervention strategies requires relevant animal models, and similar to the case for research in more advanced cancer, experimental models for early cancer fall short in recapitulating early human cancer. Therefore, the purpose of this study was to define a naturally-occurring cancer model for early detection and intervention research of translational value. This study specifically focused on high grade invasive urinary bladder cancer where there is a great need to improve patient outcomes, and where the disease is well-suited for early intervention (9, 10, 28–31).

Approximately 50% of patients with muscle invasive bladder cancer, specifically high grade invasive urothelial carcinoma (InvUC), will die from the cancer (29–31). Like many forms of cancer, InvUC is diagnosed relatively late when it is locally advanced, and in many cases has metastasized to regional and distant sites (29, 30). Higher cancer stage is a negative prognostic factor across surgical and medical therapies (29). Although interest is high in developing and testing strategies for intervening earlier in the disease course, there are limitations that are holding back this research. The numbers of patients in suitable high-risk cohorts to test screening and early intervention strategies is limited. Additionally, depending on the study design, such studies could require 15-20 years for completion (25, 30). Thus, it is essential to identify animal models that collectively represent human InvUC in the stages of cancer development, molecular and cellular features, cancer heterogeneity, aggressive cancer behavior, and host immunocompetence. While it can be challenging to collectively produce these features in laboratory animals (4–7), there is growing support for naturally-occurring canine InvUC to serve as a complementary model to experimental animal models for research, including early detection and early intervention studies (9, 28). Canine and human InvUC are notably similar in histopathology, gene expression including molecular subtypes, clinical presentation, aggressive behavior, frequent distant metastasis to lung, liver, etc., and response to chemotherapy (8–10, 28, 30, 32–35). As in humans, stage is a strong prognostic factor in dogs with InvUC (28). Interestingly, while mitogen-activated protein kinase (MAPK) pathway activation is more common in canine InvUC than in human InvUC, and is usually limited to the most aggressive human bladder cancer, the cancer in the two species converges into the same molecular subtypes (9, 10, 30, 35–38). The finding of shared luminal and basal molecular subtypes in canine and human InvUC is especially important as these subtypes have been associated with cancer behavior, treatment response, and the host immune response to the cancer (9, 10, 30, 35, 39–41).

There are additional key features of canine InvUC that are particularly relevant to early detection and early intervention research. As in humans, the cancer in dogs progresses through multiple genetic and pathologic changes including dysplasia, carcinoma *in situ* (CIS), local invasion, regional spread, and distant metastasis, with the chance to detect the disease and intervene at several different points in the cancer life cycle (28). Importantly, with the compressed life span of dogs compared to humans, a physiological period of 15-20 years in humans could be studied in 2-3 years in dogs (28).

Another particularly important feature of the naturally-occurring canine model is the very high dog breed-associated risk including a 20-fold higher risk for InvUC in Scottish Terriers (STs), and a three to six-fold higher risk in West Highland White Terriers, Shetland Sheepdogs, and beagles compared to mixed breed dogs (9, 28). This strong breed-related risk should allow early detection and early intervention studies to be accomplished with a few hundred dogs or less when focused on these high-risk subjects, whereas thousands of dogs could be required if all breeds were included (42). These collective features of canine InvUC are expected to offer unique opportunities for early detection and intervention studies of translational value once the feasibility and benefits of such studies are demonstrated (9, 28). While there are numerous published treatment-related studies in dogs presenting with lower urinary tract signs and locally advanced or metastatic bladder cancer (9, 28, 43–47), this study was very different in that it focused on screening, early cancer detection, and intervention in dogs with no clinical signs, i.e. no “symptoms” of cancer, and no outward evidence of the disease.

The purpose of this study was to determine the suitability of dogs with high risk for naturally-occurring InvUC based on their breed to serve as a relevant model for early detection and early intervention research in bladder cancer. The feasibility of this type of study, and the potential translational value as judged by similarities between the canine and human condition, were determined. There were four main components to the study. The first component was to determine the feasibility of finding bladder cancer early in dogs. While intuitively one could expect this to be possible, studies had not yet been reported to demonstrate the feasibility of early detection in dogs. The second component of the work was to assess the translational value as judged by similarities between the canine and human cancer, especially at the pathological, cellular, and molecular level. The third component was to study differences between the early and later canine bladder tumors to explore cellular and molecular changes over the course of the cancer development that could be targeted for cancer detection and intervention in the future. The fourth component of the work was to assess the results of early intervention to determine if, indeed, early cancer treatment would be as beneficial as expected and superior to the treatment of more advanced cancer. In addition to these primary endpoints, a *post hoc* evaluation of two molecularly-based exploratory urine tests was performed to explore the potential role for these tests for bladder cancer detection in future work.

In the proof-of-concept early intervention work, a cyclooxygenase (COX) inhibitor was selected for use because drugs in this class are widely used to treat canine InvUC, and have had beneficial effects in human bladder cancer in the clinical and chemoprevention setting in many, although not all, studies (9, 28, 48–50). Additionally, the availability of multiple published canine studies of COX inhibitors for comparison data (9, 28, 50, 51) was important as it would be unethical to have a non-treated control group of pet dogs with bladder cancer.

## 2 Materials and methods

### 2.1 Study overview

**Figure 1:**
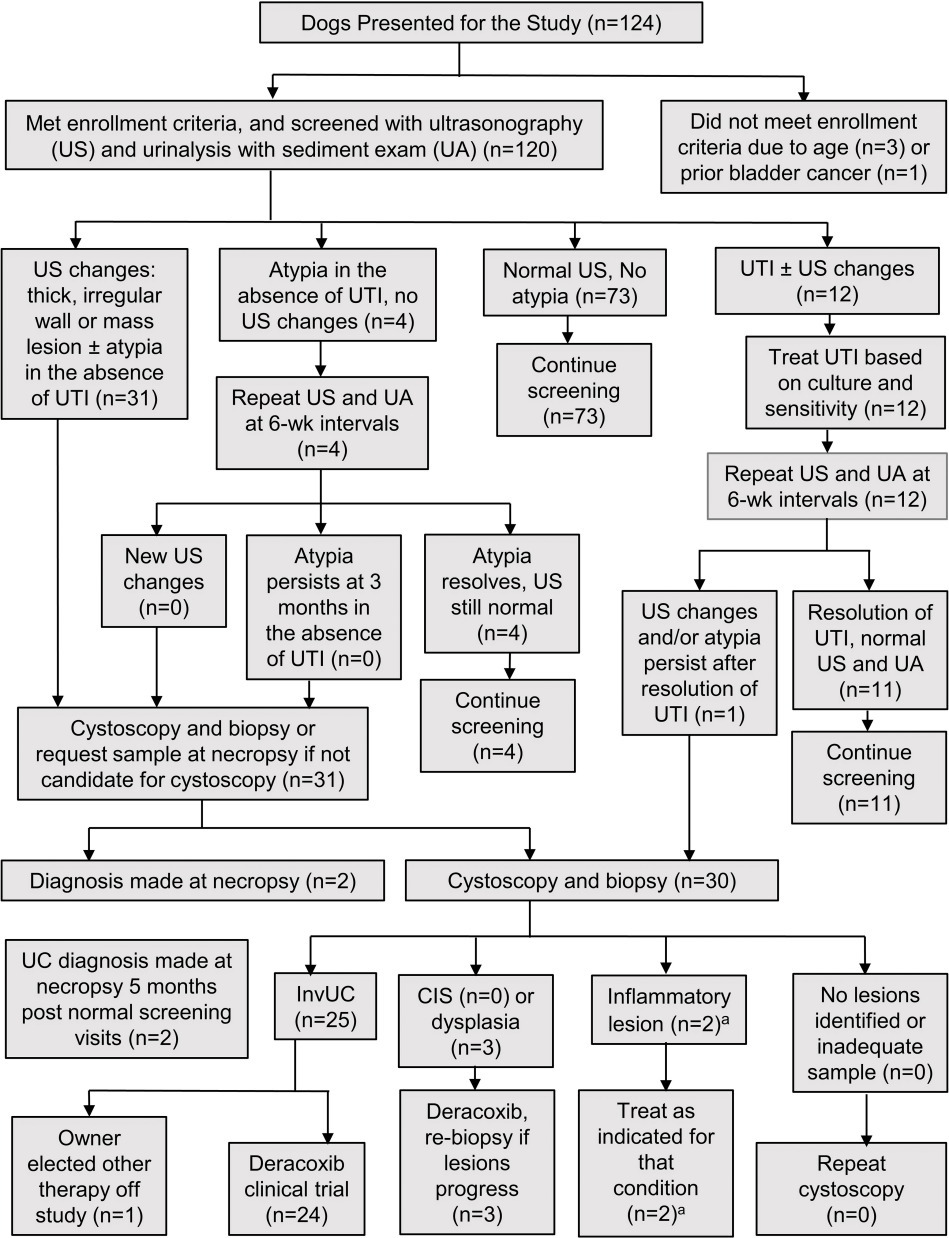
Flowchart of the screening procedures and management of dogs in the study. CIS, carcinoma *in situ*; InvUC, invasive urothelial carcinoma; UA, urinalysis with sediment exam; UC, urothelial carcinoma; US, ultrasound exam; UTI, urinary tract infection. ^a^Two dogs, 9 and 11.5 years old respectively, with thickened irregular mucosa, mass-like lesion, and epithelial atypia in urine sediment, had ulcerative cystitis identified in cystoscopic biopsy, and no evidence of progression at 16.5 and 54 months, respectively.

The study was approved by the Purdue University Institutional Animal Care and Use Committee (Protocol # 1111000124) and the Purdue University College of Veterinary Medicine Clinical Trials Review Board. In this prospective study, Scottish Terriers (STs) were screened throughout the year at the Purdue University Veterinary Hospital (PVH), West Lafayette, IN, and clinics performed by the Purdue team twice yearly at the Metropolitan Veterinary Specialty Services, Louisville, KY, and Capitol Illini Veterinary Services, Chatham, IL. The dogs continued their lives as pets in private homes. For this study, “early detection” was defined as the identification of urothelial carcinoma (UC, invasive or not), CIS, or precancerous changes, e.g. dysplasia, prior to the onset of gross hematuria, lower urinary tract clinical signs, or bladder dysfunction. Screening consisted of physical exam, ultrasonography of the urinary tract, and urinalysis, followed by cystoscopic biopsy at the PVH in dogs with positive screening tests. A flowchart of the screening procedures is provided in **Figure 1**. The clinical, pathological, and transcriptomic features of the “early” tumors were determined, and compared to: (1) published data from human InvUC, and (2) published data from STs who had not undergone screening and who were presented with lower urinary tract signs, bladder dysfunction, and more advanced (“later”) cancer, and who had not received chemotherapy, targeted drugs, or radiation therapy. Enrollment in the intervention study with the COX inhibitor, deracoxib, was open for dogs with biopsy-confirmed InvUC with the goal to compare responses between biologically early InvUC to later InvUC. Dogs with dysplasia, CIS, or noninvasive cancer that received deracoxib were evaluated separately. In *post hoc* analyses, the predictive value of two exploratory genetically-based urine tests was assessed for their potential role in bladder cancer detection in future studies (36, 37, 52). A Veterinary-Bladder Tumor Antigen (V-BTA) test for a proprietary antigen target, was performed with the expectation that the test could perform better than in previous studies of advanced UC in which hematuria caused false positives (52). A digital droplet PCR assay to detect the *BRAF^V595E^* mutation (the canine homologue of human *BRAF^V600E^*) which was described after the onset of the study, was performed at the conclusion of the study on banked samples (36, 37).

### 2.2 Eligibility, screening protocol, and follow-up

The enrollment criteria included: (1) STs ≥ 6 years old, (2) no clinical signs of urinary tract disease, (3) no prior urinary tract cancer, (4) complete resolution of any prior urinary tract infection, (5) no major organ dysfunction, and (6) written dog owner consent. Exclusion criteria included: (1) dogs in other breeds, and (2) dogs < 6 years old. There were no restrictions on sex (female, male, neutered or intact) or body weight. Screening was scheduled at 6-month intervals for 3 years including: (1) physical exam with digital rectal exam to detect thickening or masses in the urethra and prostate, and enlarged lymph nodes, (2) urinary tract ultrasonography following a standardized protocol (53) to facilitate replication of the procedure across visits, and (3) collection of a midstream free catch urine sample for urinalysis including assessment of the sediment for epithelial cell number, characteristics, and evidence of inflammation (**Figure 1**) (54). Ultrasound images were acquired of the apex, midbody, and trigone regions of the bladder, proximal urethra, and prostate in male dogs.

Cystoscopy with biopsy (55) was recommended in dogs with ultrasonographic abnormalities, persistent atypical urothelial cells of any grade, persistent hematuria in the absence of infection, or thickened areas or masses detected by physical exam. Biopsies were collected from lesions identified by mass, texture, thickness, or color. In the absence of detectable lesions at cystoscopy, the plan was to collect mucosal biopsies from the bladder apex, midbody, and trigone. Prostatic masses were sampled by cystoscopic biopsy of prostatic urethral lesions or by ultrasound-guided needle biopsy. Tissue samples were processed for formalin fixation and paraffin embedding, and for snap freezing in TRIzol (Invitrogen, Carlsbad, CA) and storage at -80˚C. The histopathology slides were reviewed by a board-certified veterinary pathologist (JRV), with tumor grade based on published criteria (28, 56). The pathologist (JRV) also assigned tumor grade to the later tumors used for comparison to the early tumors, and the pathologist was blinded as to the sample time (early *vs* later). The TNM tumor stage was assigned by WHO criteria for canine bladder tumors (57). It should be noted that the WHO classification system for canine bladder tumors differs somewhat from the classification for human bladder tumors (30, 57). In humans, T1 tumors invade the lamina propria, T2 tumors extend into the muscle, T3 tumors invade the fat layer that is deep to the muscle and below the serosa, and T4 tumors extend through the serosa or invade adjacent organs. In dogs, T2 tumors are invasive through the lamina propria and typically into the muscle layer of the bladder, and T3 tumors extend through the bladder serosa or invade adjacent organs. Extension through the lamina propria (T2) is judged in the cystoscopic biopsies, while extension through the serosa is determined by imaging studies or in surgical biopsies, if performed. Permission to perform a necropsy at the time of death from any cause was requested, but not required.

### 2.3 RNA-seq analyses

Tumor RNA was isolated, processed, and sequenced at the Purdue University Genomics Core facility, West Lafayette, Indiana as previously described (10, 35). RNA-seq analyses were performed using Strand NGS to detect differential gene expression by applying edge-R on TMM normalized data (fold change ≥2, p corr < 0.05). In subset analyses, the results from early cancer detected through screening were compared to previously published RNA-seq data from STs who were presented with more advanced InvUC, i.e., later cancer (10). The case and clinical data from these later tumors are available through the National Cancer Institute’s Integrated Canine Data Commons, ICDC study code UBC02, Accession ID 000005.

The molecular subtype of the tumors, as described in human and canine InvUC, was assigned using a 60-gene class prediction model (35), and consensus clustering (R-package Consensus Cluster Plus,1000 bootstraps, 80% subsamples, MaxK=3) was used to assess the stability of the subtype assignment. Co-expressed gene clusters in multiple groups were determined using the Clust tool (58). Functional and pathway enrichment analysis was performed using R-package ClusterProfiler (v3.18.1) and Ingenuity Pathway Analysis (IPA) toolkit. Genes predictive of T-cell-inflammation in human InvUC were used to assess the level of immune infiltration in the canine tumors (55, 59). CIBERSORT (Alizadeh Lab, Stanford University) analysis was performed using dog-human gene orthologs to estimate the abundance of specific immune cell types within the immune infiltrate (55, 60).

The comparison study between canine and human InvUC transcriptomic data was conducted in two ways. First, manual curation was used to compare the findings from the canine data to published study results from human InvUC. Second, human InvUC transcriptomic data were imported in Strand from the Cancer Genome Atlas (TCGA) (39, 61) for cross-species pathway analyses performed using multi-omic approach (Strand). Pathways that were significant in the canine screening dataset were manually curated for significant pathways in the human dataset. Data were represented by dot plots using R.

### 2.4 Deracoxib early intervention trial

The eligibility criteria for the deracoxib trial included STs with: (1) histopathologically-diagnosed, measurable InvUC detected through screening, (2) no major organ dysfunction, (3) expected survival of ≥ 6 weeks, and (4) informed dog owner consent. Dogs were excluded if they had received a COX inhibitor for more than 4 weeks in the previous year. A 10-day washout period was required for any current COX inhibitor use.

Deracoxib (Deramaxx^®^, Elanco; provided by Elanco, Greenfield IN) was administered as previously reported for dogs with InvUC (2-3 mg/kg allowed by tablet size, orally, once daily) (50). The deracoxib treatment was continued as long as the InvUC did not progress, and the drug was well tolerated. Dogs with cancer progression were eligible for other therapies off study.

The dogs were evaluated at the PVH prior to deracoxib treatment and at 6-week intervals during treatment with physical exam, CBC, serum biochemical profile, urinalysis, and urinary tract ultrasonography with mapping of tumor lesions following a standardized protocol (53, 55). Thoracic radiography and full abdominal ultrasonography to detect and measure metastases were performed at 12-week intervals. The primary tumor responses were based on volume measurements to take advantage of the most imaging information available from the ultrasound exam (53), and to allow comparison to published study data. The primary tumor responses were classified as: (1) complete remission, CR, complete resolution of all evidence of cancer, (2) partial remission, PR, ≥ 50% reduction in tumor volume and no new InvUC lesions, (3) stable disease, SD, < 50% change in tumor volume and no new InvUC lesions, or (4) progressive disease, PD, ≥ 50% increase in tumor volume or the development of new InvUC lesions (55). The response of metastatic lesions was classified by RECIST criteria (62). Adverse events were categorized using Veterinary Cooperative Oncology Group (VCOG) criteria (63).

### 2.5 Secondary endpoint data - *post hoc* analyses of urine tests for bladder cancer detection

Two secondary endpoint urine detection assays (Veterinary-Bladder Tumor Antigen Test, V-BTA; and a *BRAF^V595E^* mutation detection assay) were performed on midstream, free catch urine samples. The V-BTA test (PolyMedco Inc. Seattle, Washington, test kits provided by PolyMedco), a rapid latex agglutination urine dipstick test, was performed according to the manufacturer protocol (52) on urine samples as they were collected, and the results were analyzed at the end of the screening study. The presence of the *BRAF^V595E^* mutation was analyzed in urine sediment samples which were snap frozen and stored at -80°C over the course of the study, and the samples were run in batch at the end of the screening study. Briefly, DNA was isolated using the AllPrep DNA/RNA isolation kit (Qiagen, Germantown, MD), the presence of the *BRAF^V595E^* mutation determined by a droplet digital PCR (ddPCR) assay, and the mutation fraction, i.e., the percentage of alleles with the mutation, was determined as previously described (36, 37). Linearity in the assay was established for wild type and mutant DNA, and was preserved below 0.5 copies/µl. The sensitivity, specificity, positive predictive value (PPV), and negative predictive value (NPV) for the presence of existing disease or subsequent bladder cancer development were calculated.

### 2.6 Sample size and statistical analyses

To detect and characterize early cancer, and to assess the response to the early intervention, the goal was to identify cancer in at least 24 STs. This number of dogs is typical for phase II trials, and has been used in single arm treatment trials to estimate the response of drugs given to dogs with cancer (50, 55, 64). A minimum planned enrollment of 110 STs was based on the expectation from a ST breed health survey that 25-33% of dogs would develop bladder cancer over 3 years (65), and that early subject withdrawal during the study would be ≤ 10%.

Information recorded included age at enrollment; screening city; dog characteristics; ultrasonography, urinalyses and biopsy results; tumor location, size, and grade; and TNM stage at diagnosis and death. The data were de-identified prior to analysis.

For the deracoxib trial, the primary endpoint was remission (CR+PR). Associations between dog and tumor variables and tumor response (CR+PR *vs* SD+PD) were evaluated using logistic regression. Progression free interval (PFI, time from onset of therapy until PD) and survival times were recorded, but were considered secondary exploratory endpoints, as these could potentially be affected by lead-time bias (66). The data from dogs who were still alive and progression free at the end of the study were censored.

## 3 Results

### 3.1 Enrollment and detection of dysplasia, CIS, and UC

Of 124 STs presented for the study, 120 dogs met the enrollment criteria and participated in the study. Four dogs did not meet the enrollment criteria due to age (n=3) or prior bladder cancer (n=1). The subject demographics are summarized in **Table 1**. Of the 120 dogs enrolled, 109 dogs were screened for 3 years or until cancer development or death. The owners of 11 dogs discontinued screening early with these dogs screened: one time (two dogs), two times (one dog), three times (five dogs), and five times (three dogs), respectively.

**Table 1:** Screening site, dog features, and tumor characteristics for the dogs participating in the 3-year bladder cancer screening and early intervention study.

Characteristic	Results
Screening site	
Purdue University, West Lafayette, IN	63 dogs (53%)
Springfield, IL	33 dogs (27%)
Louisville, KY	24 dogs (20%)
Dog features	
Age at enrollment (years)	Median 9 (range 6-15)
Sex	
Intact female	7 dogs (6%)
Spayed female	66 dogs (55%)
Intact male	8 dogs (7%)
Neutered male	39 dogs (32%)
Body condition score (scale 1-9; 1 = severely underweight, 9 = morbidly obese)	Median 6 (range 5-8)
Tumor characteristics	
Size (cm^3^)	Median 1.1 (range 0.04 – 10.2)^a^
Histopathologic findings and grade where applicable	
Dysplasia (which progressed to high grade InvUC)	3 dogs (9%)^b^
Carcinoma *in situ* (high grade)	1 dog (3%)
Urothelial carcinoma, grade 1	2 dogs (6%)
InvUC, grade 2	3 dogs (9%)
InvUC, grade 3	8 dogs (25%)
InvUC, grade 4	15 dogs (47%)
TNM stage at diagnosis^c^	
T1N0M0: non-invasive	3 dogs (9%)
T2N0M0 (similar to T2-T3 in the human TNM classification system): invasive into bladder wall	27 dogs (84%)
T3N0M0 (note, these would be T4 in human TNM classification system): invasion through serosa or spread to adjacent organs	2 dogs (6%)

InvUC, invasive urothelial carcinoma; TNM, tumor node metastasis.

a For comparison, the median tumor size was 5.4 cm^3^ (range 1.2 – 36.0 cm^3^) for 18 dogs of the same breed who were already experiencing bladder dysfunction at presentation in earlier studies (10).

b These three dogs were treated with deracoxib. Progression to InvUC occurred at 7, 8, and 29 months, respectively, after detection of dysplasia.

c The WHO classification system for canine bladder tumors differs somewhat from the classification for human bladder tumors (30, 57). In dogs, T2 tumors are invasive through the lamina propria and typically into the muscle layer of the bladder, and T3 tumors extend through the bladder serosa or invade adjacent organs. In humans, T1 tumors invade the lamina propria, T2 tumors extend into the muscle, T3 tumors invade the fat layer that is deep to the muscle and below the serosa, and T4 tumors extend through the serosa or invade adjacent organs. As is typical for canine bladder cancer, the cancer involved the bladder trigone in 22 dogs, and was present in multiple sites in 14 dogs.

**Figure 2:**
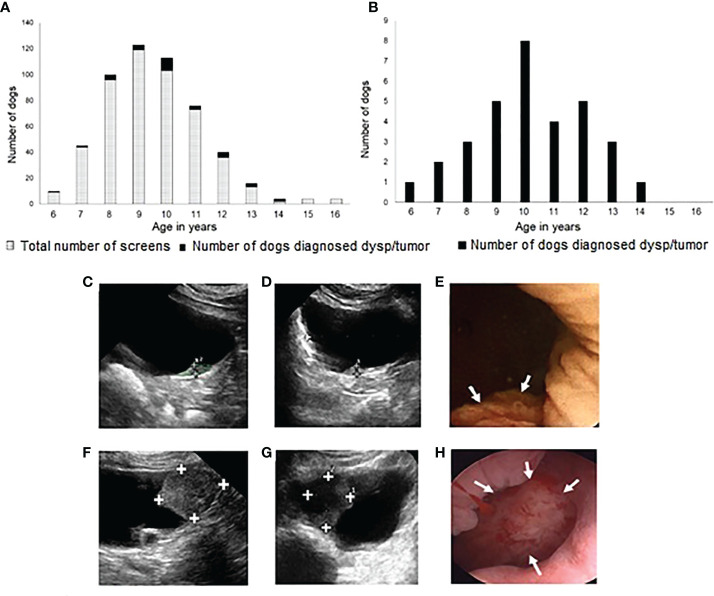
Screening history, ages of dogs screened, ages at diagnosis of bladder neoplasia, and examples of tumors detected. In **(A)**, each bar represents the number of screenings performed at each dog age during 3 years of screening. The number of dogs (Scottish Terriers, STs) diagnosed with dysplasia (dysp) or neoplasia are represented in the solid black sections at the top of each bar. Cancer was detected at the first screening visit in 13 dogs, and at later visits in 19 dogs. In **(B)**, the number of dogs diagnosed with dysplasia or neoplasia at each age are summarized. Ultrasound **(C)**, sagittal plane, **(D)**, transverse plane**)** and cystoscopy **(E)** images are included of a 0.3 x 0.3 x 0.5 cm early luminal subtype invasive urothelial carcinoma (InvUC) detected through screening. A small area of mucosal proliferation was visible at cystoscopy **(E**, arrows**)**. For comparison, images are included from a ST with later luminal subtype InvUC not detected through screening **(F-H)**. A 1.6 X 2.3 X 1.9 cm mass occupies most of the trigone region of the bladder in the ultrasound sagittal plane **(F)**, transverse plane **(G)**, and cystoscopy **(H**, arrows**)** images.

In the absence of any urinary tract signs, 32 dogs had bladder cancer detected and diagnosed by biopsy during 3 years of screening (**Figures 1**, **2**). The dog and tumor characteristics, and TNM stage information are summarized in **Table 1**. The number of dogs screened and number of tumors detected at each age, and representative ultrasound and cystoscopy images are provided in **Figure 2**.

The histopathological diagnosis was made by cystoscopic biopsy in 28 dogs and at necropsy in four dogs. The findings leading to cystoscopy included: bladder mass and epithelial atypia (17 dogs), bladder mass and no atypia (eight dogs), bladder wall changes and atypia (one dog), and bladder wall changes and no atypia (two dogs). Hematuria was uncommon with five dogs with cancer having > 4 RBCs/HPF at the time of diagnosis. Four of the dogs with cancer had proteinuria of 2+ or above. Of the four dogs with the cancer diagnosis made at necropsy, two dogs had abnormal ante mortem ultrasound exams but did not undergo cystoscopy due to comorbid conditions (metastatic melanoma in a dog with grade 3 InvUC; hepatic abscess in a dog with CIS). The other two dogs died from peliosis hepatis and hepatocellular carcinoma, respectively, and screening tests had been normal at 5 months and at 5.5 months prior to death; both dogs had low grade UC. Other bladder lesions detected during screening included a suspected anatomic variant in STs consisting of a small nonprogressive apex nodule in seven dogs (67), and biopsy-confirmed ulcerative cystitis in two dogs (**Figure 1**). Ultrasound changes of masses or thick irregular bladder wall in the absence of infection were noted in six additional dogs, but the owners of those dogs declined biopsy.

### 3.2 Transcriptomic findings

#### 3.2.1 Characteristics of the early canine cancer

**Figure 3:**
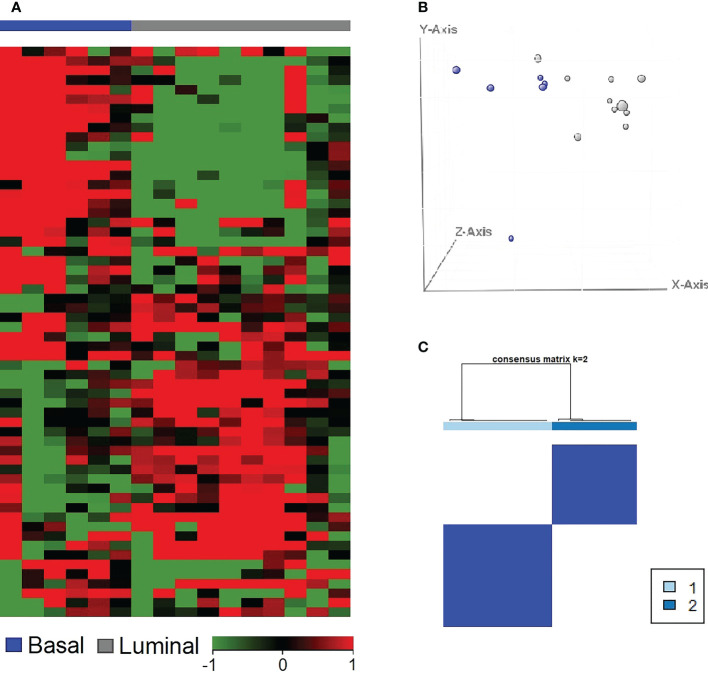
Basal and luminal subtypes present in canine early InvUC samples similar to those in human InvUC. A previously published class prediction model (35) was used to assign subtype to the canine InvUC samples. Of the 16 tumors, 10 tumors (gray) were luminal, and six tumors (blue) were basal **(A)**. The results were confirmed in PCA plot **(B)**, and in consensus clustering **(C)**. The stability of the groups was confirmed by consensus clustering with 94% accuracy. InvUC, invasive urothelial carcinoma; PCA, principal component analysis.

**Figure 4:**
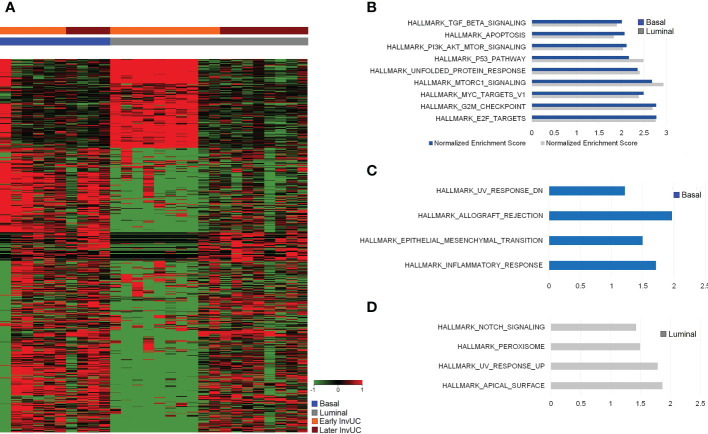
RNA-seq and Gene Set Enrichment Analyses (GSEA) of early canine invasive urothelial carcinoma (InvUC) demonstrating shared and subtype-specific pathways of importance in human bladder cancer. In the heatmap **(A)**, each column represents data from one “early” sample, and the samples are grouped according to luminal or basal molecular subtype (see key), with expected heterogeneity observed within and between samples. Differentially expressed genes (n=2785, FC ≥2, pcorr ≤ 0.05) were identified that were unique to early InvUC as compared to normal bladder mucosa from dogs with no urinary disease. Enriched gene sets (Hallmark collection) in early basal and early luminal InvUC were identified with GSEA and depicted with Normalized Enrichment Score (NES) **(B-D)**. In addition to shared gene sets between the molecular subtypes, such as *TGFβ, TP53, PI3K-AKT-mTOR* signaling, and apoptosis pathway **(B)**, marked differences between the subtypes were readily apparent **(A, C, D)**. Inflammatory Response, Allograft Rejection, and Epithelial-Mesenchymal Transition gene sets were enriched in early basal tumors **(C)**, and *Notch* signaling, UV-Response Pathway, and Apical Surface gene sets were enriched in early luminal tumors **(D)**. Additional genes and pathways identified in Ingenuity Pathway Analyses are summarized in **Table 2**.

**Figure 5:**
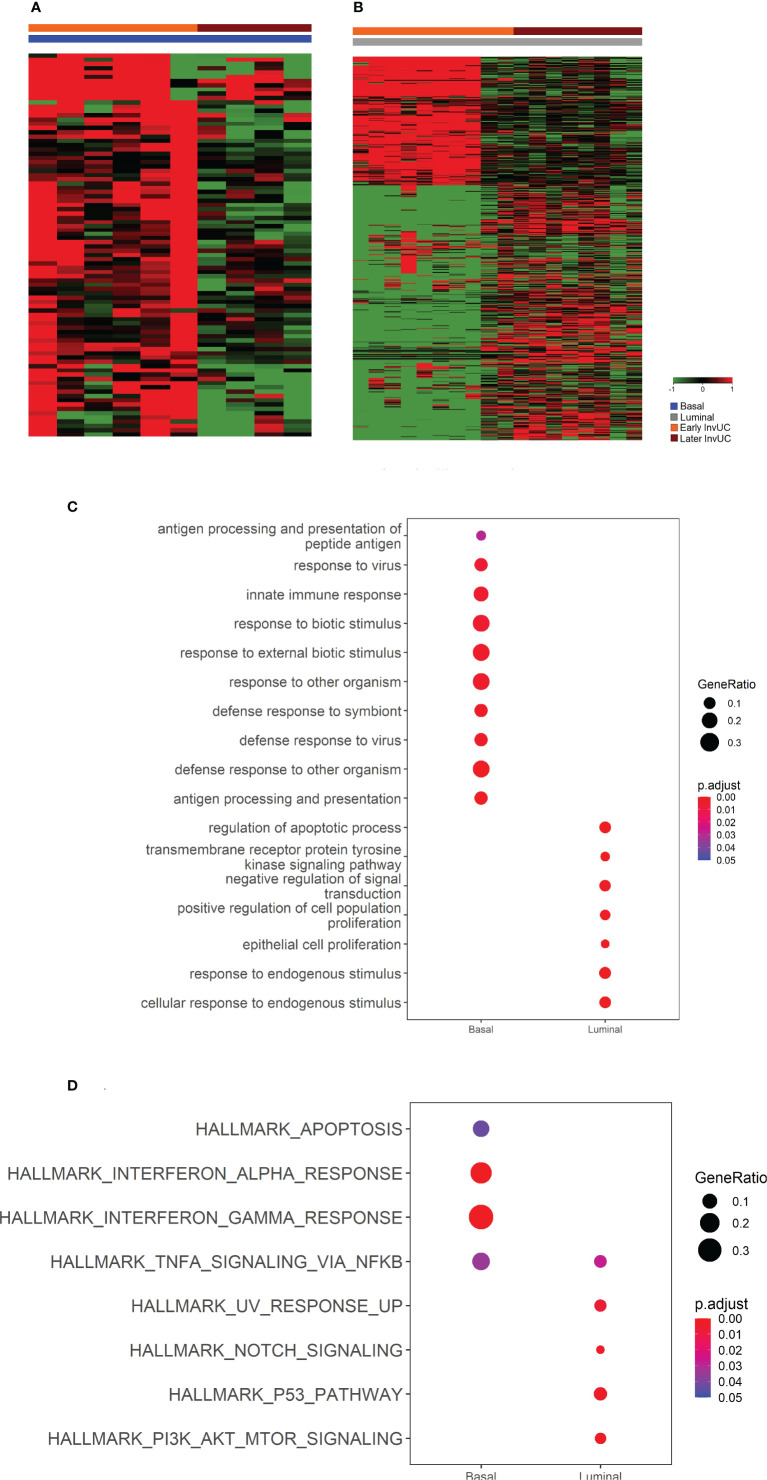
RNA-seq and Gene Set Enrichment Analyses (GSEA) demonstrating differences between early and later canine invasive urothelial carcinoma (InvUC) within each molecular subtype. This part of the study was done to search for genes that differed in expression between early and later tumors. In the heatmaps **(A, B)**, each column represents data from one sample, and the samples are grouped according subtype and time of detection (see key). CompareClust was utilized to identify Gene Ontology (GO) terms **(C)** and Hallmark pathways **(D)** that differed between the early and later tumors within each subtype. The gene ratios from the GO and Hallmark pathway analyses are indicated by the size of the dot, and the *P* value is indicated by color scale in the key **(C, D)**. Substantial differences were found between early luminal and later luminal tumors including GO terms related to proliferation and apoptosis, and Hallmark pathways related to proliferation such as *PI3K-AKT-mTOR* and *TP53* pathways. In Ingenuity Pathway Analysis, the later luminal tumors also had upregulation of Cell Cycle Control of Chromosomal Replication, *CDK, EGF, FGFR1, NRAS*, and *RRAS* genes, and downregulation of *HIPPO* signaling and Cell Cycle G1/S Checkpoint Regulation (**Table 2**). Differences between the early and later basal tumors were limited, but when present included GO terms and Hallmark pathways related to apoptosis, and the immune response.

**Figure 6:**
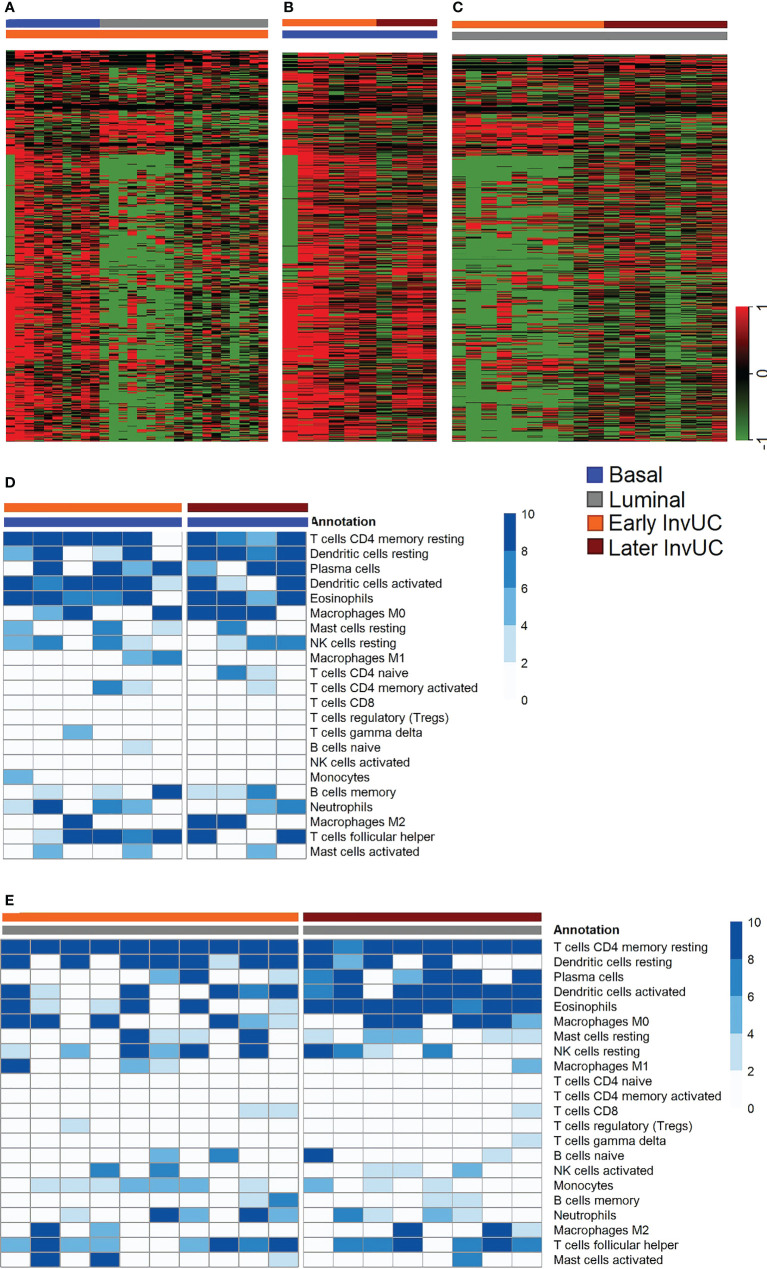
RNA-seq immune signatures in early and later canine invasive urothelial carcinoma (InvUC). Previously reported genes known to predict T-cell inflammation in human InvUC were used to identify immune infiltration (immune hot) or lack of immune infiltration (immune cold) signatures in early and later canine InvUC **(A-C)**. The heatmaps in **A-C** were constructed to denote cold immune signatures in green, and hot signatures in red (52). Note that early basal tumors were much more immune hot than early luminal tumors **(A)**. Early and later basal tumors were similar in immune infiltration signatures **(B)**. Later luminal tumors were less immune cold than early luminal tumors **(C)**, but were still less hot than basal tumors. The expected heterogeneity is observed within and between samples. In CIBERSORT analyses, the types of immune cells responsible for the immune signatures were similar between subtypes and time points **(D, E)**.

Tumor tissues for RNA-seq analyses were available from 16 dogs with InvUC, including 10 luminal and six basal tumors (**Figure 3**) (10, 30, 35). Enriched gene sets that were shared between the early luminal and the early basal tumors included *TGFβ, TP53, MYC, PI3K-AKT-mTOR* signaling, and apoptosis and DNA repair pathways. In addition to shared genes, there were marked differences in other differentially expressed genes between early luminal and early basal tumors (**Figures 4**, **5**). Genes, for example, related to the inflammatory response, allograft rejection, and epithelial-mesenchymal transition were enriched in early basal tumors, whereas *Notch* signaling, UV-response pathway, and apical surface gene sets were enriched in early luminal tumors. Additional pathways identified in Ingenuity Pathway Analysis are included in **Table 2**. In other transcriptomic findings, the signatures of immune infiltration were greater in the early basal tumors than in the early luminal tumors (**Figure 6A**).

**Table 2:** Results of Ingenuity Pathway Analysis (IPA), examples of pathways with a significant –log p-value (> 1.3) and a Z score of ≥ 1.5.

Condition	Examples of pathways and genes of interest
Pathways and genes shared between early luminal and early basal subtype tumors as compared to normal canine urothelium	Upregulation of Base Excision Repair Pathway, Kinetochore Metaphase Signaling Pathway, and SPINK1 Pancreatic Cancer Pathway; and *NRAS*, *RRAS, RAC1, CD44, MAP2K2, R2RL1, and SMAD2* genes Downregulation of Phagosome Formation, and Calcium Signaling; and *CCR* and *CXCR* family genes
Other pathways and genes differentially expressed in early basal tumors as compared to normal canine urothelium	Upregulation of Ephrin Receptor Signaling, Endothelin 1 Signaling, Cell Cycle Control of Chromosomal Replication, CXCR4 signaling, signaling by Rho Family GTPases; and *AKT3, CREB3* and *5, EGF, EGFR1, EPHA3* and *8, RASA1, RASD1* and *2, ACTA2, IFNAR1, VEGB-D, FGF1, IL1-5, IL-10, IL-13, IL-20, IL-27, VIM, REL, RELA*, and *RASD1 genes*
Other pathways and genes differentially expressed in early luminal tumors as compared to normal canine urothelium	Upregulation of Kinetochore Metaphase Signaling, and Polyamine Regulation in Colon Cancer Pathways; and *CENPA, DNAH1, H2AX, KNL1, RAD21, STAG3, ZWILCH, ZWINT, AKT1 and 3, ARAF*, and *MYC* genes.
Pathways and genes that were altered (higher IPA Z-score) in later basal tumors compared to early basal tumors	Upregulation of Epithelial Adherens Junction Signaling, SPINK1 Pancreatic Cancer Pathway, Role of MAPK Signaling in Promoting the Pathogenesis of Influenza, and *AKT3, EGF, FGF1, FGFR1, RRAS*, and *HGF* genes. Downregulation of Phagosome Formation and CREB Signaling in Neurons
Pathways and genes that were altered (higher IPA Z-score) in later luminal tumors compared to early luminal tumors	Upregulation of Cell Cycle Control of Chromosomal Replication, and Epithelial Adherens Junction Signaling, and *CDC6* and *45, CDK2,6,14, CDT1, MCM6, ORC1* and *6, PRIM2, EGF, FGF1, FGFR1, HGF, NRAS, RAC1, RASD1* and *2, RRAS, CCL5* and *10* genes in later tumors compared to early tumors. Downregulation of HIPPO signaling, Cell Cycle G1/S Checkpoint Regulation, and G-Protein Coupled Receptor Signaling pathways in later tumors compared to early tumors.

IPA, Ingenuity Pathway Analysis.

#### 3.2.2 Similarities between the canine and human cancer

**Figure 7:**
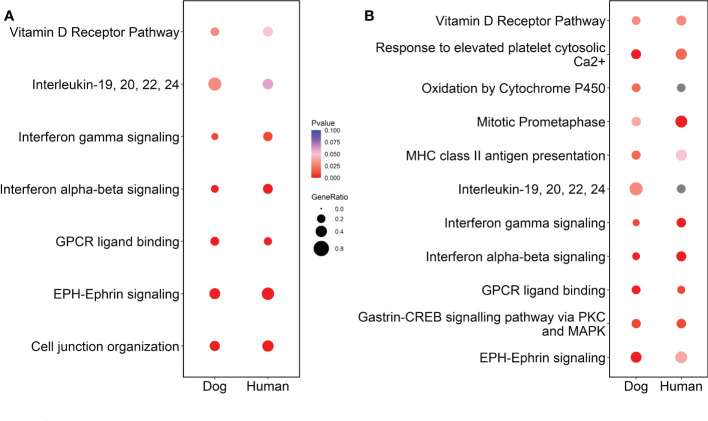
Cross-species comparison of RNA-seq data from the early tumors in dogs and human InvUC data from TCGA. Cross-species comparison was conducted using multi-omic pathway analyses in Strand and represented as dot plots using R. Pathway analyses were conducted concurrently using differentially expressed genes in the early canine tumors detected through screening (as compared to normal) and high grade human tumors stage T1-T3 (as compared to normal) in panel **(A)** or to human tumors stage T1-T2 in panel **(B)**. With the finding that the majority of the canine tumors had not yet penetrated beyond the muscle layer, the comparison to human T1-T2 tumors was of interest. Note, the WHO classification for canine bladder tumors differs somewhat from the classification for human bladder tumors (30, 57). In dogs, T2 tumors are invasive through the lamina propria and typically into the muscle layer of the bladder, and T3 tumors extend through the bladder serosa or invade adjacent organs. In humans, T1 tumors invade the lamina propria, T2 tumors extend into the muscle, T3 tumors invade the fat deep to the muscle and below the serosa, and T4 tumors extend through the serosa or invade adjacent organs. Significant pathways (p<0.05) were selected and compared across species in both analyses groups. Note the similarities in pathways between the canine and human tumors, along with expected differences in some. Interestingly, more relevant pathways were noted when the dog data was compared to human T1-T2 than when compared to human T1-3 data.

Considerable similarities in gene expression and pathways, as well as expected differences, were noted between the early canine tumors and human InvUC. Key findings are summarized in **Table 3** and **Figure 7**.

**Table 3:** Examples of pathways shared between canine bladder cancer detected through screening and human invasive bladder cancer (30, 39, 44, 45, 68–74).

*MAPK* *Notch* *PI3K-AKT-mTROR* *EGF/EGFR* *TP53* *MYC*	*E2F* target G2M checkpoint Apoptosis DNA repair including Base Excision Repair Kinetochore Metaphase Signaling

#### 3.2.3 Comparison between early and later canine cancer

The dogs with early tumors (n=16) were similar to the comparison group of STs with later tumors (n=12) in age, sex, tumor location, and grade (**Table 4**) (10). Marked transcriptomic differences between the early and later tumors were noted, especially between early luminal and later luminal tumors including gene ontology (GO) terms for proliferation and apoptosis, and Hallmark pathways such as *PI3K-AKT-mTOR* and *TP53* pathways (**Figure 5**). The later luminal tumors also had upregulation of cell cycle control of chromosomal replication, *CDK, EGF, FGFR1, NRAS*, and *RRAS* genes, and downregulation of *HIPPO* signaling and cell cycle G1/S checkpoint regulation in Ingenuity Pathway Analysis (IPA) (**Table 2**). Differences between the early and later basal tumors were not as common, but included GO terms and Hallmark pathways related to apoptosis, and the immune response (**Figure 5**; **Table 2**).

**Table 4:** Dog and tumor characteristics associated with tumor samples used in RNA-seq analyses including “early” tumors detected through screening, and “later” tumors detected in dogs presenting with lower urinary tract clinical signs and bladder dysfunction.

Parameter	Early tumors	Later tumors^a^
Number of dogs (1 tumor/dog)	16	12
Dog breed	16 STs	12 STs
Age at diagnosis (yrs), median (range)	9.7 (6.3-12.9)	9.6 (7.8-15.0)
Sex		
Spayed female	10 dogs	7 dogs
Intact female	1 dog	2 dogs
Neutered male	5 dogs	2 dogs
Intact male	0 dogs	1 dog
Body weight (kg)	11.2 (range 9.0-13.8)	11.5 (7.2-17.2)
Prior chemotherapy or radiation therapy	0 dogs	0 dogs
Prior COX inhibitor treatment	3 dogs	3 dogs
Tumor location		
Tumor in trigone and/or urethra	12 dogs	8 dogs
Tumor limited to apex and/or mid bladder	4 dogs	4 dogs
TNM stage		
T2N0M0	14 dogs	12 dogs
T3N0M0	2 dogs	0 dogs
Tumor volume (cm^3^), median (range)	1.6 (0.1-5.4)	4.3 (1.2-36.0)
Tumor grade		
Grade 2	1 dog	1 dog
Grade 3	4 dogs	3 dogs
Grade 4	10 dogs	8 dogs
Grade not available	1 dog	0 dogs
Tumor subtype		
Luminal	10 dogs	8 dogs
Basal	6 dogs	4 dogs

COX, cyclooxygenase; STs, Scottish Terriers.

a These STs were part of a larger study including dogs across multiple breeds (10). Additional information concerning the STs and their tumors, including clinical data and tumor RNA-seq data, is available in the National Cancer Institute’s Integrated Canine Data Commons (ICDC study code UBC02, Accession ID 000005).

In other transcriptomic findings, immune infiltration (immune hot) patterns persisted between the early and later basal tumors (**Figure 6B**). In luminal tumors, the immune signatures were cold in the early tumors and increased somewhat in the later tumors compared to the early tumors, but the later luminal tumors were still more immune cold than the basal tumors (**Figure 6C**). In CIBERSORT analyses, the types of immune cells responsible for the immune signatures were similar between the subtypes and time points (**Figures 6D, E**). Heterogeneity, as expected, was observed between samples, and immune features, such as upregulation of M2 macrophages and immune checkpoint genes were observed in some of the individual dog samples.

### 3.3 Deracoxib early intervention trial

Twenty-four STs with InvUC were enrolled in the deracoxib trial including 15 female dogs (14 spayed, one intact dog) and nine male dogs (eight neutered, one intact dog). The dogs had a median age of 10.2 years (range 6.3-13.9 years), median body weight of 10.4 kg (range 9.0-15.7 kg), and median tumor size of 1.0 cm^3^ (range 0.04-10.2 cm^3^). Two dogs had stage T3N0M0 tumors (equivalent to T4 human tumors), and 22 dogs had T2N0M0 tumors (similar to T2-T3 human tumors) (30, 57).

The deracoxib was well tolerated. Two dogs did accidentally ingest their entire deracoxib prescription from their most recent PVH visit (42 X 25 mg deracoxib chewable tablets) at one time. Both dogs received emergency care by the local primary care veterinarian including induction of emesis, and neither dog had any detected ill effects. The development of syncope 13 months into deracoxib treatment in one dog, and retinal atrophy at 5 months into treatment in another dog were considered unrelated to deracoxib administration.

**Figure 8:**
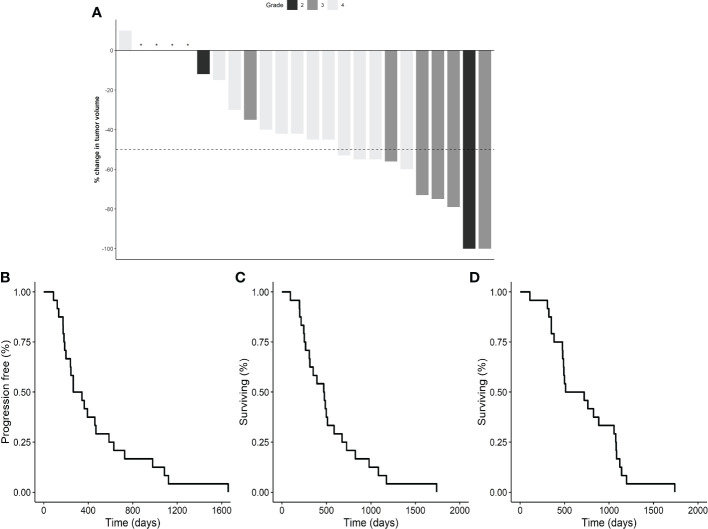
Deracoxib early intervention results. A waterfall plot of the percentage change in tumor volume in individual dogs treated with deracoxib is provided in panel **(A)**. Responses included: CR in two dogs (8%), PR in eight dogs (33%), and SD in 14 dogs (58%). The gray scale denotes tumor grade (see key). Kaplan-Meier survival curves are included for progression free interval (PFI) **((B)**, median 304 days**)**, deracoxib-related survival **((C),** median 473 days**)**, and overall survival **((D),** median 615 days**)**. Following deracoxib failure, eight dogs received intravenous chemotherapy (carboplatin, vinblastine, mitoxantrone), and seven dogs received oral therapies (chlorambucil, toceranib, piroxicam). Distant metastases were present in 26% of dogs at death. Six dogs died from non-urinary cancer related causes. CR, complete remission, no clinically detectable cancer remaining; PFI, progression free interval; PR, partial remission, ≥ 50% reduction in tumor volume; SD, stable disease, < 50% change in tumor volume. "*" indicates no change in tumor volume.

The 42% remission rate (8.3% CR, 33.3% PR) compared very favorably to the 17-25% remission rate with COX inhibitors in more advanced canine InvUC (9, 28, 50, 51) (**Table 5**; **Figure 8**). In exploratory endpoints, the median progression free interval (PFI) was 304 days, the median deracoxib-related survival was 473 days, and the median overall survival was 615 days (**Figure 8**). Distant metastases were present in 26% of the dogs at death. The dogs were more likely to have remission if the InvUC was located in the trigone (nine of 15 dogs, 60%) than away from the trigone (one of nine dogs, 11%) (OR 12.0, 95% CI 1.18 – 122.27, *P*=.013). Dogs with grade 4 cancer had shorter PFI than dogs with grade 2 cancer (HR 6.1, 95% CI.79 – 47.02, *P*=.007). Older age at enrollment was associated with shorter survival (HR 1.8, 95% CI 1.25 - 2.72, *P*=.001).

**Table 5:** Results of cyclooxygenase (COX) inhibitor treatment in STs in the screening study and in dogs with later more advanced bladder, i.e. “symptomatic” cancer in other published studies (28, 50, 51)^a^.

Treatment	Number of dogs	Remission (CR+PR) rate (%)	Median PFI (days), and (range)	Median survival (days), and (range)
Deracoxib in STs in the screening study (current study)	24	42	304 (87-1659)	615 (109-1739)
Deracoxib in dogs across breeds presented with more advanced cancer (previous study) (50)	26	17	133 (36-482)	323 (25-696)
Piroxicam in dogs across breeds presented with more advanced cancer (previous study) (28)	76	21	120 (17-1256)	244 (6-1256)
Firocoxib in dogs across breeds presented with more advanced cancer (previous study) (51)	12	25	101 (20-313)	146 (8-739)
COX inhibitors (three studies above) given as frontline therapy to dogs across breeds presented with more advanced cancer (28, 50, 51)	83	20	125 (20-1243)	243 (8-1256)
COX inhibitors (three studies above) given as frontline therapy to STs presented with more advanced cancer (28, 50, 51)	16	21	120 (56-312)	310 (81-588)

COX, cyclooxygenase; PFI, progression free interval, time from the onset of treatment until cancer progression; ST, Scottish Terrier.

a These studies were performed at the same institution as the screening study and followed similar methods in assessing treatment response.

### 3.4 Exploratory urine detection tests

The two exploratory urine detection tests had frequent false positive and negative results, and low predictive value (**Table 6**). The positive predictive value, for example, was only 33% for the V-BTA test, and 44% for the *BRAF* mutation test (**Table 6**).

**Table 6:** Summary of findings from exploratory molecularly-based urine tests including a Veterinary-Bladder Tumor Antigen Test (V-BTA) (52) and a ddPCR assay to detect the presence of the *BRAF^V595E^* mutation (36, 37).

Condition	Number of dogs tested	Sensitivity	Specificity	PPV	NPV
V-BTA test results and predicting the presence of urothelial carcinoma					
All dogs tested, test positive, yes/no	112^a,b^	74%	41%	33%	80%
Dogs undergoing necropsy, test positive, yes/no	37	64%	42%	70%	90%
Urine *BRAF^V595E^* mutation and predicting the presence of urothelial carcinoma					
*BRAF^V595E^* detected, yes/no	112^b,c^	87%	55%	44%	92%
*BRAF^V595E^* MtF ≥ 2%	112^b,c^	78%	84%	66%	90%
Dogs undergoing necropsy, *BRAF^V595E^* detected, yes/no	37	85%	50%	82%	56%
Dogs undergoing necropsy, *BRAF^V595E^* MtF ≥ 2%	37	70%	60%	83%	43%

ddPCR, droplet digital polymerase chain reaction; MtF, mutation fraction; NPV, negative predictive value; PPV, positive predictive value; V-BTA, Veterinary Bladder Tumor Antigen.

a The V-BTA test (PolyMedco Inc. Seattle, Washington) (52) was performed on 434 urine samples available from 118 dogs, i.e. samples available after urinalyses had been performed. The test was positive in 15 of 22 dogs with grade 3-4 cancer, five of five dogs with grade 1-2 cancer, three of three dogs with dysplasia, three of six dogs with suspect cancer, and 47 of 81 dogs with no dysplasia or cancer detected over a median of 23 months followup.

b In calculating these parameters, results from dogs with suspect tumors and no biopsy were excluded as it was not possible to know whether to classify these as tumor-bearing or non-tumor-bearing dogs.

c The test was performed on 383 urine sediment samples available from 117 dogs to detect the presence of *BRAF^V595E^*, the dog homologue of *BRAF^V600E^*. *BRAF^V595E^* was detected in 22 of 23 dogs with grade 3-4 cancer, four of five dogs with grade 1-2 cancer, two of three dogs with dysplasia, three of five dogs with suspect cancer, three dogs that later developed cancer, and in 33 of 80 dogs with no bladder dysplasia or neoplasia detected over a median of 18 months followup. The MtF varied from < 1% to > 90% (median 43.0%), and was very low (< 2%) in 28 dogs. Additionally, when the test was repeated in dogs undergoing treatment, there was no association between the MtF or changes in MtF and the response to deracoxib.

## 4 Discussion

The study results provide strong evidence that dogs with high breed-associated risk for bladder cancer can serve as a valuable translational model for early detection and early intervention research. Canine studies are expected to complement and extend the work in experimental models. An initial key finding of this study was the feasibility of detecting early disease, i.e. dysplasia, CIS, or UC which was still small, organ-confined, and “asymptomatic” in 27% of the dogs during 3 years of screening. As discussed below, the screening criteria can be adjusted as needed to study larger numbers of dogs, and to study and intervene at different times in the disease process. The work also increased the understanding of early cancer and the similarities to the human condition, and provided evidence that drugs can, indeed, be more effective in the early disease setting. The evidence for the translational value of the canine model and the relevance to human cancer included the similarities between the canine and human tumors in pathologic features; transcriptomic patterns including molecular subtypes, immune patterns, signaling pathways, and genes; and clinical behavior (9, 10, 28, 30, 39).

In the pathologic analyses of the canine tumors detected by screening, > 80% of the tumors were grade 3 and 4 InvUC, similar to other reports of canine bladder cancer (9, 28). It is well recognized that different molecular pathways drive the development of low grade superficial bladder tumors which are more common in humans, compared to high grade invasive bladder cancer, and it is likely that the pathways driving low grade tumors are not active in most dogs (9, 28, 30). The findings in the dogs in this study were also consistent with prior reports that high grade cancer does not typically start with a low grade precursor, rather it is high grade when first detected (30). These finding again demonstrate that canine bladder cancer is best suited as a model for high grade invasive bladder cancer, the most serious form of the cancer in humans where advances are especially needed.

The transcriptomic findings highlighted several pathways and genes of interest in the early canine tumors that are important in human InvUC including *TP53, TGFβ, MYC, PI3K-AKT-mTOR*, and DNA repair signatures (39, 68–71). Similarly, in the cross-species analyses, pathways related to mitotic activity, IFNα, IFNβ, IFNγ, and EPH-Ephrin signaling were shared between the canine and human tumors. The transcriptomic analyses within subtypes were particularly informative. Early basal tumors already had upregulation of multiple pathways driving oncogenic signaling, cell proliferation, progression, pro-angiogenic activity, and immune cell infiltration, and these signatures persisted in the later basal tumors consistent with the aggressive nature of basal tumors in humans and dogs (10, 39–41). In contrast, upregulated pathways in early luminal tumors were more limited, but were noticeably more prevalent in later luminal tumors, including upregulation of *MAPK, Notch, PI3K-AKT-mTOR, EGF*, and *TP53* pathways, and downregulation of *HIPPO* signaling, G-Protein Coupled Receptor Signaling, and Cell Cycle G1/S Checkpoint Regulation, all of which have been associated with the development and progression of human bladder cancer (30, 39, 40, 68–74). Importantly, these findings indicate that while basal tumors have activation of multiple oncogenic pathways early in the disease course, that luminal tumors can be detected when pathway activation is still much more limited, offering expanded research opportunities in cancer development, progression, and intervention.

The transcriptomic analyses of the immune signatures were also very informative. This is especially important since many of the antibodies and immune assays typically available for human and rodent studies, do not exist for canine samples. The transcriptomic immune signatures differed substantially between the molecular subtypes of the canine tumors. Building on earlier work in dogs and humans presenting with “symptomatic” cancer (10, 40, 41), the canine basal tumors were immune infiltrated, i.e. immune hot, and this occurred even in the early disease setting. Early luminal tumors were largely immune cold. Although the later luminal tumors were less immune cold, these tumors were still less immune infiltrated than basal tumors, consistent with findings in human InvUC (39–41). In CIBERSORT analyses, the lack of significant differences in immune cell types between early and later tumors was not surprising. The immune makeup had not yet been influenced by chemotherapy, targeted drugs, radiation, or innate effects over the extended life of the cancer (26, 75). While immune exhaustion signatures which are often acquired later in the disease course were largely absent, immune suppressive features such as upregulation of M2 macrophage and immune checkpoint genes (26, 75) were already present in the early tumors in a small number of individual dogs. With the rapid growth of immunotherapy in human InvUC, yet the dire need to improve upon this therapy approach, the opportunity to study dogs with cold and hot immune signatures similar to those in humans could prove to be highly valuable (29).

The results of the early intervention trial with deracoxib aligned well with the expectation that earlier cancer would be more responsive to treatment. The 42% remission rate (CR+PR) in the dogs from the screening study compared very favorably to the remission rates of 19% in STs and 17-25% in dogs across breeds in published studies of single-agent COX inhibitors in more typically-advanced InvUC (9, 28, 50, 51) (**Table 5**). Dog owners also appreciated the excellent quality of life in their dogs. Stranguria, hematuria, and pollakiuria, typical of canine InvUC, remained absent unless substantial cancer progression occurred. The distant metastatic rate at death in the screened dogs (26%) also compared favorably to the 52-58% distant metastatic rate in dogs presented with more advanced InvUC in previous studies, as did the exploratory endpoint data on PFI and survival (**Table 5**; **Figure 8**) (9, 28, 50). The response to deracoxib was not associated with COX-2 expression in the tumor. This was not surprising. While deracoxib predominantly inhibits COX-2, some COX-1 inhibition could occur with the doses used (76), and these and other effects independent of COX-2 could potentially enhance the antitumor activity of the deracoxib (9, 28, 77, 78).

It is important to note that future early detection and intervention studies in dogs can be tailored to meet specific study goals. Based on the enrollment in previous canine clinical trials, approximately 25% of the 40,000 to 60,000 dogs that newly develop bladder cancer each year in the United States are expected to be in high-risk breeds, indicating plenty of dogs for screening and early intervention studies (8, 9, 28, 50, 55). While the 6-month screening interval was sufficient here as there were no dogs that were normal at one visit and had large or metastatic cancer at the next, the screening interval could be shortened and cystoscopy performed using standard or targeted approaches (79, 80) at the onset of any changes to potentially detect more dogs with dysplasia and CIS for future studies. Primary prevention studies could commence at an earlier age, again noting that a physiological time period of 20 years in humans could be studied in 3 years in dogs (28). While the exploratory molecular-based urine detection tests in this study had low predictive value, as further molecular targets for detection are identified, and new tests for bladder cancer are developed and validated, these could also have a role in early detection, and potentially allow detection of dysplasia and CIS in more dogs. When considering the results from a different perspective, however, further research related to the urine detection tests used in this study is warranted to determine the causes of the false positive results, and the mechanisms that could potentially prevent the emergence of clinically-detectable cancer in dogs with the molecular markers detected in their urine. There is a future vision for more widespread bladder cancer screening in dogs in high-risk breeds. Expanded screening would be beneficial to the dogs, and could provide an ongoing source of dogs to participate in early intervention studies.

Different types of therapies can be evaluated in the early disease setting in future studies building on the initial proof-of-concept work with deracoxib. The selection of deracoxib for the initial work was considered appropriate based on the: (1) availability of comparison data from multiple published trials in dogs with more advanced cancer, recognizing that having a non-treated control group of dogs with InvUC would be unethical, (2) acceptance of COX inhibitors as part of most treatment protocols in canine InvUC (9, 28, 43, 50), (3) excellent safety profile and low risk of side effects of deracoxib (50), especially important for dogs that were not experiencing cancer-related clinical signs, and (4) evidence of the beneficial effects of COX inhibitors in human bladder cancer (48). In the future, however, a variety of drugs could be evaluated in the early disease setting including immunotherapies, chemotherapies, and targeted drugs. There is considerable interest in learning how the response to immunotherapies such as immune checkpoint inhibitors can be enhanced according to the time in the tumor immunity cycle (26), and how to convert immune cold tumors to immune hot tumors to improve their responsiveness to therapy. Dogs could offer this opportunity for study as the cancer can be detected at early stages, and it is accepted to give promising new therapies in a treatment naive setting to dogs before the dogs have failed multiple other therapies (8, 9, 28). Targeted drugs could also be evaluated in the context of different levels of signaling pathway activation. Examples of the types of targeted drugs that could be tested include *mTOR* inhibitors recognizing that the *PI3K-AKT-mTOR* pathway is active in human and canine InvUC, that *mTOR* inhibitors have had at least modest activity in more advanced human InvUC, and that drugs in this class have been evaluated in dogs with other types of cancer (39, 81, 82). Similarly, *EGFR* and *HER2* are overexpressed in human and canine InvUC, and drugs targeting these molecules have had *in vivo* activity in canine and human InvUC trials (44, 45, 83, 84). There is considerable interest in learning if the administration of these drugs earlier in cancer development will be more effective. In addition to utilizing the canine model for early intervention studies, there is also interest in applying the canine model to address other critical questions in human bladder cancer through research designed to: (1) gain a broader understanding of the mechanisms of bladder cancer development and progression, and the identification of new treatment targets, (2) understand the role of environmental and host factors in cancer development and therapy, (3) identify new markers of early cancer to facilitate early detection, and (4) assess the value of circulating tumor DNA and other liquid biopsy strategies (9, 28).

In conclusion, the results of this study support the feasibility and translational value of early detection and early intervention research in dogs with high breed-associated risk for InvUC. This will build on the previous and ongoing use of canine InvUC in treatment studies involving dogs with locally advanced or metastatic cancer (8, 9, 28). The study also demonstrated similarities between InvUC in dogs and humans, and defined expected differences in cellular and molecular processes between early cancer detected through screening and later more advanced cancer. The clinical, pathological, and transcriptomic data will be available in the National Cancer Institute’s Integrated Canine Data Commons to allow other scientists to test new hypotheses, thus greatly expanding the value of the study. Importantly, related to drug development, the work provided evidence for the feasibility of early intervention trials in dogs, and evidence that drugs applied earlier in the cancer process can, indeed, be more effective than when applied to more advanced cancer. These outcomes establish a foundation for including dogs in high-risk breeds for InvUC in studies to better understand bladder cancer development and progression, to develop and test early intervention strategies, and to ultimately improve the outlook for humans and dogs facing bladder cancer.

## Data availability statement

The datasets presented in this study can be found in online repositories. The names of the repository/repositories and accession number(s) can be found in the article/supplementary material.

## Ethics statement

The animal study was reviewed and approved by the Purdue University Institutional Animal Care and Use Committee and the Purdue University College of Veterinary Medicine Clinical Trials Review Board. Written informed consent was obtained from the dog owners for the participation of their animals in this study.

## Author contributions

All authors contributed to the design, methods, and acquisition of data. The initial draft of the manuscript was written by DK, DD, SU, and AR. All authors reviewed and edited the manuscript, and approved the submission.

## Funding

Funding was provided by the Scottish Terrier Club of America, and private donations made for this research study at Purdue University. The work was also facilitated by the Purdue Genomics Core, and the Collaborative Core for Cancer Bioinformatics supported by the Purdue University Center for Cancer Research NIH grant number P30 CA023168 and the Indiana University Simon Cancer Center NIH grant number P30 CA082709, and the Walther Cancer Foundation, Indianapolis, IN. Elanco, Greenfield, IN, donated deracoxib for the intervention trial. PolyMedco Inc., Seattle, Washington donated test kits for the Veterinary-Bladder Tumor Antigen test.

## Acknowledgments

The authors thank the clinicians and staff of the Purdue Comparative Oncology Program for their excellent work on this study. The authors thank Dr. Byron McCall, Ms. Lisa Hills, and the staff of Capitol Illini Veterinary Services, Ltd, Chatham, IL; and Drs. Randall Graehler and Catherine Daley, Ms. Rose Shacklett, and the staff of Metropolitan Veterinary Specialists, Louisville, KY for allowing the Purdue team to hold screening clinics in their practices, and for supporting the work there. The authors gratefully acknowledge the Scottish Terrier Club of America, regional breed clubs, the Door County WI Scottie Rally and similar events, and the work of Dr. Marcia Dawson for helping identify dogs for the study, and facilitating their enrollment and follow-up. And, the authors sincerely thank all of the families and their Scottish Terriers for making the study successful.

## Conflict of interest

The authors declare that the research was conducted in the absence of any commercial or financial relationships that could be construed as a potential conflict of interest.

## Publisher’s note

All claims expressed in this article are solely those of the authors and do not necessarily represent those of their affiliated organizations, or those of the publisher, the editors and the reviewers. Any product that may be evaluated in this article, or claim that may be made by its manufacturer, is not guaranteed or endorsed by the publisher.
